# Changes in Physical Fitness in Youth Padel Players during One Season: A Cohort Study

**DOI:** 10.3390/sports12070193

**Published:** 2024-07-16

**Authors:** Sofia Ryman Augustsson, M. Charlotte Olsson, Emma Haglund

**Affiliations:** 1 Department of Sport Science, Faculty of Social Sciences, Linnaeus University, SE-392 31 Kalmar, Sweden; 2 Department of Environmental and Bioscience, School of Business, Innovation and Sustainability, Halmstad University, SE-301 18 Halmstad, Sweden; charlotte.olsson@hh.se (M.C.O.); emma.haglund@hh.se (E.H.); 3 Spenshult Research and Development Centre, Bäckagårdsvägen 47, SE-302 74 Halmstad, Sweden

**Keywords:** isometric squat strength, jump performance, sit-ups, lateral agility, padel training

## Abstract

The aim of this study was to investigate how physical fitness performance, which is related to the strength and power of the lower extremities and core, as well as lateral agility, changes across 20 weeks of in-season training in youth female and male padel players. This study was conducted using a prospective cohort design on 16 Swedish high school padel players aged between 15 and 18 years old. The players were assessed at baseline with five tests of their physical fitness and followed prospectively, with the registration of their training load for 20 weeks, and then assessed at a follow-up, approximately five months later. The players increased their performance in all tests (*p* ≤ 0.02). The mean improvement in their Isometric squat test performance was 20% for peak force, 18% for relative strength value and 69% for average rate of force development. Their improvement in the squat jump test performance was 9%, whereas the improvement in their countermovement jump test was 6%. For the 30 second sit-up test, an improvement of 14% was observed. Improvements were also noted for the 30 second side hop test performance on both the right (9%) and left leg (11%). The effect size ranged from 0.31 to 1, respectively, for the tests, representing a small to large effect. The data from this study suggest that an improvement in physical fitness performance can be obtained during 20 weeks of padel training.

## 1. Introduction

Padel is considered the fastest growing sport in the world, with more than 10 million people playing the sport [1]. There are approximately 100 associations in Sweden that together currently amount to ~20,000 members [2]. Hence, padel has grown into a very popular sport and probably plays an important role in promoting physical activity while limiting sedentary behavior. Padel combines elements from tennis and squash and is almost always played in doubles. The court is about one-third the size of a tennis court and is surrounded by glass walls, making the game more intense and creating a unique playing environment. The ball is allowed to hit the surrounding walls before landing on the opponent’s court, adding an element of unpredictability to the game [3]. The unique material composition and surface texture affect how the balls interact with the court surface, resulting in variations in bounce and trajectory. The small size of the ball allows for a faster game that requires quicker reactions, than, for example, tennis. Thus, padel involves rapid changes in direction; frontal and lateral changes in position; and rotations, including repetitive and high-speed movements of the upper extremities. The duration of World Padel Tour (WPT) matches is approximately 90 min [4] and advanced padel players seem to cover an average of 3000 m per match [5]. However, it has been observed that the distance covered is dependent on variables such as the level of play, where players at higher levels appear to cover greater distances [5]. There are a lot of both defensive and offensive tactics in padel, which refer to positioning, the types of strokes and shots used and the combination of play. For example, proper positioning is crucial for effective defense in padel, while taking control of the net through aggressive net play is a key offensive tactic in padel. In professional men’s padel, groundstrokes are the most used game actions, followed by volleys, and the largest number of actions are carried out with a ball coming directly from the opponent and in the net zone [6]. There are more actions performed on an upward trajectory (53.9%) compared to a downward trajectory (46.1%) [6].

Furthermore, there are variables that have been noted as predictors of performance: stroke type, net distance, shot direction and trajectory [6]. Most winning shots in padel are hit from the net area, with smashes being the most effective technique for scoring points among professional players [5]. Men tend to win more points with flat and topspin smashes, while women prefer the Bandeja (a shot between a smash and a forehand volley) [5]. Additionally, cross-court shot trajectories are generally more successful in winning points or causing opponent errors compared to down-the-line shots [5]. However, it is also important to consider that the performance in padel is not only about the technical or tactical skills of the game itself, but also associated with the level of physical conditioning. Due to the high-intensity nature of the game, cardiorespiratory factors, strength and agility are considered important factors in optimizing padel performance [5]. For example, padel players need sufficient strength and power in their lower body and core to execute powerful shots and withstand the physical demands of the game. In addition, padel requires quick reactions and fast movements to cover the court effectively. Thus, players need agility to respond to the fast-paced nature of the game, as well as speed to reach and hit the ball in time, and lateral agility is an essential variable [7].

In recent years, there has been increasing interest in studying the sport of padel by investigating game variables [8,9,10], physical performance [11,12] and injury panorama [13,14,15]. However, the literature is scarce regarding studies on youth players. A growing number of players are found among young players aged between 14 and 18 years old and there are currently over 800 licensed youth players in Sweden [2]. Thus, padel is well established among young people, but research that describes the physical fitness and training load in young players is limited. Only a few studies have been conducted on the physical performance of youth padel players [8,12,16]. In one study, match activity was studied in young padel players, which was characterized by longer rallies, longer rest intervals and more strokes per rally compared to other racket sports, which resulted in lower effort [8]. In addition, U-16 male players have a shorter total playing time and fewer strokes per game compared to U-18 players [8]. In another study where physical performance was assessed in youth players, it was observed that throwing strength was influenced by practice experience [12]. However, this study was completed with a cross-sectional design and the athletes were not followed prospectively. These findings reinforce the importance of understanding the sport’s unique playing characteristics to optimize training accordingly.

As more and more young people play organized padel, it is important to obtain deeper knowledge regarding their physical fitness to ensure greater sports participation and performance in this group of athletes. It has been argued that there is a lack of clarity regarding physical preparation and athletic fitness for padel athletes and that it would be desirable to investigate this through physical fitness from a longitudinal perspective [17]. To date, no prospective study seems to exist on physical fitness performances in youth padel and how in-season training changes these characteristics. Thus, the aim of this study was to investigate how physical fitness performance, related to the strength and power of the lower extremities and core, as well as lateral agility, changes across 20 weeks of in-season training in youth female and male padel players.

## 2. Materials and Methods

### 2.1. Study Design and Participants

This study was conducted, using a prospective cohort design, on 16 Swedish high school padel players aged between 15 and 18 years old (Table 1). The players were recruited through the Swedish sports high school system. Eighteen players fulfilled the inclusion criteria and performed tests at baseline. Two were excluded from the analyses due to drop-out at follow-up. The inclusion criteria were female and male registered padel players, aged 15–19 years old, with at least 1 year of experience. Players with any pain/discomfort at the test sessions that could influence their performance were excluded. Prior to inclusion, players were informed of the benefits and risks of participating and signed an informed consent form.

### 2.2. Procedure

Players were assessed at baseline in October 2023 with five tests of their physical fitness and followed with a weekly registration of their training load for 20 weeks. The players were assessed approximately five months later, in March 2024, using the same tests. The tests were conducted by five experienced test leaders and took place on Monday mornings at the Human Movement Lab, Halmstad University. Anthropometrics (height, weight, body composition (total body lean body mass (LBM)) were measured at the beginning of the test session and before the tests of their physical performance.

### 2.3. Assessment of Muscle Function and Physical Performance

The methods used for the tests are described elsewhere. A summary including references is provided below. The test battery aimed to assess both the strength and power of the participants’ lower extremities and core, as well as muscular endurance and lateral agility, as these characteristics are essential to the physical demands of the game [5,7].

#### 2.3.1. Lower Extremity Muscle Strength

The maximal muscle strength of the lower extremities was measured with the Isometric squat test [18], including the relative strength value, expressed relative to bodyweight (N/kg bodyweight), and average rate of force development (avgRFD). The player held a static squat position, with their knees at 45° angles, pushing down, with maximal effort, into a force plate [19] for ~5 s. Their peak isometric force (N) and avgRFD (N·s^−1^) were recorded and assessed using a force plate and analyzed with commercial software (MuscleLab, Ergotest Technology AS, Langesund, Norway). Three maximal efforts were performed with three minutes of rest in between trials and the best attempt was used for further analysis. The avgRFD was calculated from the best peak force value. High test–retest reliability (ICC = 0.97) and validity (r = 0.97) have previously been noted for the Isometric squat test [20].

#### 2.3.2. Power and Jump Ability

Muscular power in the lower extremities was evaluated with jump tests: the squat jump (SJ) and countermovement jump (CMJ) [11]. A photoelectric vertical jump system (IVAR Test system, SH sport & fitness, Mora, Sweden) was used for both tests, as descried in [21]. IVAR is an infrared system with a transmitter, receiver and connected clock which collect the data [22]. The transmitter and receiver were placed on the floor with a 150 cm spacing. The IVAR system then measured the flight time from standing to landing and transferred that to jump height in centimeters. The best of three attempts was used for further analysis. High test–retest reliability has been observed for both the SJ and CMJ (Cronbach’s alpha = 0.97 and 0.98 respectively), as well as great validity (r = 0.81 and r = 0.87 respectively) [23].

#### 2.3.3. Core Strength

Core strength was assessed with the 30 second sit-up test, as previously described [24]. The maximum number of repetitions of sit-ups, performed with maximum speed for 30 s, was documented. The 30 second sit-up test has previously been shown to have a high test–retest reliability (ICC_2,2_ = 0.93) [24].

#### 2.3.4. Lateral Agility and Muscular Endurance

The lateral agility and muscular endurance of the lower extremities were evaluated with the 30 second side hop test for each leg, as described [25]. The maximum number of repetitions was documented. The 30 second side hop test has been shown to have excellent validity (ICC = 0.93) and reliability (ICC = 0.92) [26]. The aim of using the side hop test for the assessment of lateral agility was also to evaluate any between-limb differences.

### 2.4. Anthropometry

Body height (cm) was measured with a stadiometer, and body mass (kg) was obtained from the bioelectrical impedance analysis (BIA) measurement (InBody 770, InBody Co, Ltd., Seoul, Republic of Korea), together with total body lean body mass (LBM). The measurements were performed with the players having fasted overnight, barefoot, dressed in light clothes, and in a position according to the standard procedure recommended by the manufacturer. A trained research technician performed the measurements and the results were used based on accompanying InBody 770 software analyses. InBody770 has been noted as reliable for measuring the fat free mass in healthy adults (ICC ≥ 0.99) [27].

### 2.5. Training Regimes

**Figure 1:**
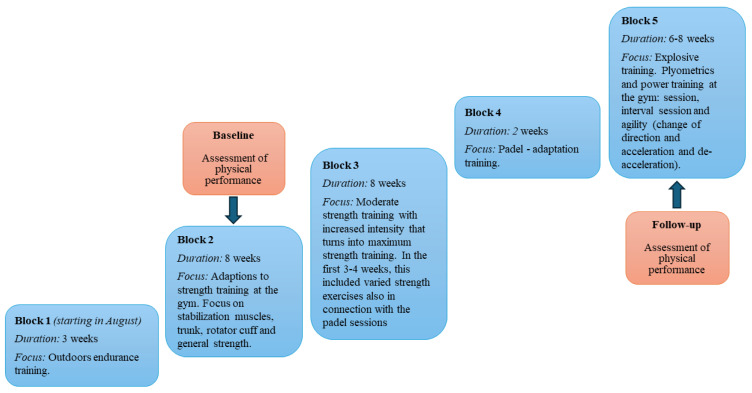
Training regimes during the padel season.

The training and game regimes were as follows: Players attended coach-supervised training for three padel sessions and two strength and conditioning sessions per week. Additionally, the players performed unsupervised training, usually performed as one padel session and one strength and conditioning session per week. The players also took part in games during weekends. The supervised sessions consisted of five blocks (Figure 1).

### 2.6. Statistical Analysis

Statistics were calculated using IBM SPSS (IBM SPSS Statistics for Windows, Version 29.0. IBM, Armonk, NY, USA). Descriptive data are presented as means and standard deviation (SD). For a comparison of the baseline vs. follow-up, as well as of between-limb differences, the data were analyzed using the Paired sample t-test, with a 95% CI. The LBM value is expressed in kg. The average RFD (avgRFD) was calculated by dividing the peak force by the time to achieve peak force (Peak Force [N]/Time to achieve peak force [s]). The effect size was calculated to determine the magnitude of the in-season training (padel and conditioning training) effect using the following formula: Pre-post effect size = posttest mean − pretest mean/pretest SD [28]. A scale for determining the magnitude of the effect size in trained players was used, which identified <0.25 as representing a trivial effect, 0.25–0.5 as a small effect, 0.50–1.0 as a moderate effect and 1.0 or greater as a large effect [28]. The level of significance was set at *p* < 0.05. Based on the results from a previous study [29] investigating the influence of in-season training on physical fitness performance, we hypothesized that there would be at least 4% difference in performance from baseline to follow-up for all tests. The estimated number of participants required to achieve a power of 0.80 (α = 0.05) was 14. Therefore, this study was planned to recruit a minimum of 17 padel players considering potential dropouts.

## 3. Results

The training loads (hours of training and games) are given in Table 2 and the test performance values for male and female players are given in Table 3. The players increased their performance in all tests and their LBM from baseline to follow-up (*p* ≤ 0.02) (Table 4). The mean improvement in their Isometric squat test performance was 20% for peak force (*p* = 0.002), 18% (*p* = 0.003) for relative strength and 69% for avgRFD (*p* = 0.003). Their SJ test performance increased by 9% (*p* = 0.005) and their CMJ by 6% (*p* = 0.021). For the 30 second sit-up test, a mean improvement of 14% (*p* = 0.004) was observed. Improvements were also noted in the 30 second side hop test performance on both the right (9%, *p* = 0.01) and left leg (11%, *p* = 0.001), with no between-limb differences at either baseline (*p* = 0.715) or at follow-up (*p* = 0.152). The effect size ranged from 0.31 to 1 for the tests, representing small to large effects.

## 4. Discussion

To our knowledge, this is the first study investigating the influence of training on physical performance in youth padel players. The present study provides new data on physical performance and in-season changes in youth padel players.

### 4.1. Improvements in Physical Fitness Performance

The greatest improvement was noted for the Isometric squat test, where an increased peak force of 20% and avgRFD of 69% were observed. A large effect size of 1.00 or greater was also noted, confirming an important improvement. For relative strength, the improvement was somewhat lower but still showed a moderate effect size. To our knowledge, the Isometric squat test has not previously been used in research on padel, and squat strength tests of any kind are also limited. However, in one study on elite male table tennis players, the one-repetition maximum (1RM) barbell squat test was used to evaluate the effect of weightlifting or plyometric training for eight weeks on physical performance [30]. In this study [30], the 1RM strength increased by 20% in the weightlifting and 14% in the plyometric group. These improvements are in line with the findings in the present study. Still, the sample consisted of players of a different sport and the intervention was only eight weeks long [30]. In addition, the 1RM barbell squat strength test is used to evaluate isotonic strength, whereas maximal strength was assessed isometrically in the current study. Isotonic testing is argued to be most similar to the characteristics of sports [31,32], whereas isometric tests differ from the dynamic nature of most sports [32]. Yet, we believe that the 1RM barbell squat test may be harmful if the player is inexperienced with the test and it may not measure maximum strength due to errors in technique. The isometric squat test is safer and also generates data on the RFD when using a force plate and is therefore a compelling test for measuring athletic performance [18]. The RFD is considered to be an important aspect of athletic performance in sports where the time to develop force is limited (e.g., sprinting and jumping) [33]. Thus, the use of the Isometric squat test with a force plate is most likely a relevant test for padel athletes. In addition, the relatively large improvement in avgRFD in relation to the moderate improvement in peak force noted in the current study suggests that the training regimes targeted athletic performance efficiently.

Although improvements were achieved in both jump tests, the effect size was small. However, the jump results in the present study (SJ ~35 cm, CMJ ~37 cm) are greater compared to what has previously been observed in youth padel athletes [12], and also compared to earlier data on male elite players (SJ ~27 cm, CMJ ~33 cm) [11]. In addition, the effects of a 6-week explosive strength training program on the physical performance of amateur female padel players (aged 25–40) were previously investigated [34]. The CMJ value noted (~21.5 cm) in this study [34] is lower compared to the values noted for the female players in the present study, both in the pre- (25 cm) and post-test (28 cm). The great jump values noted in the present study suggest that high school padel players are highly trained. They also indicate that elite padel sport is still in a highly developmental phase and that the elite players of the future most likely will have greater fitness and, thus, greater athletic performance than those today. However, there seems to be a greater disparity (6 cm) between performance in the SJ and CMJ in elite padel players [11] compared to the group of youth players (3 cm) in the current study. This may be due to maturation and jump experience. Functional performance slowly improves with maturation [35] and the full development of a specific skill depends on the full maturation of the nervous system, which happens approximately at the age of 20 [36]. Adult elite athletes most likely have adaptations from chronic exposure to stretch–shortening cycle (SSC) actions. Hence, the greater CMJ performance, compared to the SJ, noted in the study on elite players [11] may probably be due to their greater technique and better utilization of elastic forces [37]. As the players in the current study consisted of adolescent athletes, they probably still have several years to develop to reach their full potential for the use of SSCs. Still, any age-related differences in enhanced performance could not be demonstrated in the current study due to the restricted sample size.

For core strength, a mean improvement of 14% in the 30 second sit-up test was observed, which is lower compared to a previous study evaluating the effect of a 26-week in-season strength training program in young female volleyball players [29]. In that study, the performance in the 30 second sit-up test was increased by 55% [29]. However, the number of sit-ups performed by volleyball players [29] is inferior compared to the present study, both in the pre-test (11 versus 22) and at post-test (17 versus 25). Still, the participants in the volleyball study consisted of female athletes, whereas the subjects in our study were mostly male athletes (*n* = 14). Moreover, the number of sit-ups (25) recorded at the follow-up is almost as high as what has previously been observed in firefighters [38] (28 repetitions). Still, instead of 30 s, the test was performed for one minute. Hence, our findings regarding sit-up performance suggest that the players in the current study are highly trained athletes.

Improvements were also noted for their 30 second side hop test performance. The side hop test is commonly used to evaluate between-limb differences [25] and the return-to-sport decision after ACL injury [39] and measures hop endurance and lateral agility [25]. As far as we know, the test has not previously been used to assess physical fitness performance in padel. However, the importance of performance in lateral displacement and split-step in padel has previously been demonstrated [7]. Thus, the side hop test may be a valuable test for determining lateral agility in padel. Yet, different aspects of validity related to specific padel agility performances need to be further addressed. The mean number of repetitions in the present study (~60) is somewhat higher than earlier presented normative values [25,26,40]. In addition, the players in the present study did not display any between-limb differences. These findings suggest that the players had great symmetrical hop endurance and agility, which may be due to their participation in padel training as well as a strength and conditioning program.

Regarding LBM, only a minor increase was observed from baseline to follow-up, which may be due to maturation rather than to specific training regimes. However, the LBM noted at baseline (~33 kg) is in line with what has previously been noted for adult male tennis (~33 kg) and padel players (~32 kg) [41]. Thus, the players in the present study can be considered an athletic sample. InBody770 has been noted as reliable in both men and women for measuring fat free mass (ICC ≥ 0.99) [27]. However, a systematic bias (overestimation of FFM) has been noted when the agreement between InBody and DXA has been investigated [27]. In addition, in one previous study, a bioelectrical impedance analysis using InBody 770 was compared to whole-body DXA scans in male and female college athletes [42]. The study revealed a significant difference in total body fat free mass by 1.6 kg between BIA and DXA (BIA: 59.1 kg; DXA 57.5 kg) and suggested that BIA may not be accurate in measuring athletic populations [42]. Still, DXA and BIA have been shown to equally track increases in whole-body FFM, and BIA has been suggested as an acceptable alternative to DXA for estimating the total FFM changes in athletes [43].

Approximately ~16 h of training and games per week per player were observed, representing a relatively high training load. This training load (~12 h) is similar compared to what has previously been noted for elite padel players (~11 h per week) and higher than that noted for sub-elite players (~6 h per week) [44]. In contrast, this training load is lower compared to what has previously been noted in another study on elite players, where there mean training hours were ~24 h per week [11]. A maximum of 48 h of training and games were noted during one week for one of the players in the present study. This must be considered an extremely high training load and may have contributed to the great increase in physical fitness performance seen during the season.

This study provides insight into physical fitness and training in youth padel players, which may be valuable in the in the work of identifying performance factors and in the development of strength and conditioning programs, as well as youth talent development programs. This study indicates that youth high school padel players seem to have great physical fitness, which may be due to specialized training and professional coaching. These indications also suggest that padel is still in a largely developing phase, with youth players exhibiting a physical fitness similar to what has previously been noted for elite players. It is likely that we will see a transformation in professional padel in the coming decades as more younger talents emerge in the sport. Thus, it may also be interesting to follow lifestyle factors to describe this group of athletes and investigate how these factors may influence changes in their physical fitness more clearly.

### 4.2. Strengths and Limitations

The main strength of the present study is that the sample consisted of a homogeneous group of youth athletes within the same age range in a high school padel program. The players took part in the same training regimes, and they all had experience of the extra training load that comes with attending a sports high school. However, there are some limitations to the present study. First, the results may be affected by the small sample size, especially the small sample of female players. For this reason, the data were analyzed for the entire group and not broken down by sex. In a previous study, it was noted that game demands were significantly influenced by sex in youth padel players [8]. In contrast, in another study [12], the impact of sex on physical fitness was rather small and only jumping ability differed between female and male players. However, this study also consisted of a rather small sample (*n* = 34) [12]. Thus, sex differences in physical fitness need to be further investigated in a larger cohort of youth padel players. Second, the players were assessed first thing on a Monday morning due to the player’s schedule, and all players had been participating in games during the weekend. The players were most likely fatigued to some degree and their performance may have been affected. However, the tests were carried out at the same time at both test sessions, and all the players had experienced an extra match load from the previous weekend. Lastly, maturity data were not collected, which is a factor that might have influenced the results. Physical fitness and sports performance gradually improve with maturation [35]. Body size increases and muscle mass and strength develop, which differ between the sexes [45]. Thus, future studies are recommended to investigate how maturation status influences the in-season performance and physical fitness of youth female versus male padel players.

## 5. Conclusions

Data from this study suggest that increases in physical fitness performance can be obtained during 20 weeks of padel training. The findings also indicate that Swedish high school padel players are highly trained, most likely due to their specific strength and conditioning training in addition to regular padel training sessions. The test battery used in this study can also be recommended to assess in-season changes in physical fitness in youth padel players. Future studies may be conducted to confirm these results in a larger group of players at different levels of play and ages.

## Figures and Tables

**Table 1:** Players’ characteristics at baseline.

Variables	Females (*n* = 3)	Males (*n* = 15)	Total Cohort (*n* = 18)
	Mean (SD)	Mean (SD)	Mean (SD)
Age (year)	16.7 (0.4)	16.9 (0.9)	16.8 (0.9)
Height (cm)	171.5 (4.4)	180.5 (7.0)	179.0 (7.4)
Weight (kg)	65.4 (11.1)	67.4 (8.0)	67.1 (8.2)

**Table 2:** Mean hours of training and games, presented per week (*n* = 16).

	Mean (SD)	Min–Max
Specific padel training	7.3 (2.8)	2–20
Total training	11.9 (4.3)	2–35
Game	4.3 (1.9)	0–18
Total training and games	16.2 (7.0)	2–48

**Table 3:** Results of the physical performance tests for female (*n* = 2) and male (*n* = 14) players at baseline and at follow-up.

Test Variables	Male Players at Baseline	Male Players at Follow-Up	Female Players at Baseline	Female Players at Follow-Up
	Mean (SD)	Mean (SD)	Mean (SD)	Mean (SD)
The Isometric squat test (N)	1307 (286)	1564 (310)	1158 (45)	1444 (49)
Relative squat strength (N)	19.5 (4.4)	22.8 (4.7)	19.6 (0.8)	23.8 (0.9)
avgRFD (N·s^−1^)	577 (343)	1011 (388)	668 (289)	854 (298)
SJ (cm)	33.6 (5.2)	36.6 (5.7)	24.1 (2.5)	26.0 (3.8)
CMJ (cm)	36.5 (5.5)	38.5 (5.7)	25.0 (5.4)	28.4 (4.9)
30 second sit-ups (No)	22 (4)	25 (3)	25 (5)	26 (6)
30 second side hop (R), (No)	57 (8)	63 (4)	39 (2)	42 (2)
30 second side hop (L), (No)	56 (7)	64 (5)	48 (3)	57 (1)
LBM (kg)	34.2 (3.4)	34.8 (3.1)	25.6 (0.7)	26.5 (0.0)

avgRFD: average rate of force development; SJ: squat jump; CMJ: countermovement jump; (R): right; (L): left; LBM = total body lean body mass.

**Table 4:** Results of the physical performance tests for the total cohort (*n* = 16).

Test Variables	Baseline	Follow-Up	Difference	Effect Size
	Mean (SD)	Mean (SD)	Mean (CI)	
The Isometric squat test (N)	1289 (260)	1549 (292)	260 (114–407) *	1.00
Relative squat strength (N)	19.5 (4.1)	23 (4.4)	3.5 (1.4–5.5) *	0.85
avgRFD (N·s^−1^)	588 (329)	992 (373)	403 (155–652) *	1.23
SJ (cm)	32.4 (6.4)	35.3 (6.5)	2.9 (1.1–4.8) *	0.45
CMJ (cm)	35.1 (6.8)	37.2 (6.5)	2.1 (0.4–3.8) *	0.31
30 second sit-ups (No)	22 (4)	25 (3)	3 (0.9–4.2)	0.75
30 second side hop (R), (No)	55 (9)	60 (8)	5 (2–10) *	0.55
30 second side hop (L), (No)	57 (7)	63 (5)	6 (4–9) *	0.86
LBM (kg)	33.2 (4.4)	33.8 (4.1)	0.6 (0.1–1.1) *	0.14

avgRFD: average rate of force development; SJ: squat jump; CMJ: countermovement jump; (R): right; (L): left; LBM = total body lean body mass; * = significant difference from baseline to follow-up.

## Data Availability

The data presented in this study are available on request from the corresponding author due to ethical restriction.
